# Current views on selenoprotein S in the pathophysiological processes of diabetes-induced atherosclerosis: potential therapeutics and underlying biomarkers

**DOI:** 10.1186/s13098-023-01247-y

**Published:** 2024-01-03

**Authors:** Shan-shan Yu, Jian-ling Du

**Affiliations:** 1 Department of Endocrinology and Metabolism, The First Affiliated Hospital of Dalian Medical University, Dalian, 116011 Liaoning China; 2 Dalian Key Laboratory of Prevention and Treatment of Metabolic Diseases and the Vascular Complications, Dalian, 116011 Liaoning China

**Keywords:** SELENOS, Atherosclerosis, Diabetes mellitus, Single nucleotide polymorphism (SNP), Therapeutics and biomarkers

## Abstract

Atherosclerotic cardiovascular disease (ASCVD) consistently ranks as the primary mortality factor among diabetic people. A thorough comprehension of the pathophysiological routes and processes activated by atherosclerosis (AS) caused by diabetes mellitus (DM), together with the recognition of new contributing factors, could lead to the discovery of crucial biomarkers and the development of innovative drugs against atherosclerosis. Selenoprotein S (SELENOS) has been implicated in the pathology and progression of numerous conditions, including diabetes, dyslipidemia, obesity, and insulin resistance (IR)—all recognized contributors to endothelial dysfunction (ED), a precursor event to diabetes-induced AS. Hepatic-specific deletion of SELENOS accelerated the onset and progression of obesity, impaired glucose tolerance and insulin sensitivity, and increased hepatic triglycerides (TG) and diacylglycerol (DAG) accumulation; SELENOS expression in subcutaneous and omental adipose tissue was elevated in obese human subjects, and act as a positive regulator for adipogenesis in 3T3-L1 preadipocytes; knockdown of SELENOS in Min6 β-cells induced β-cell apoptosis and reduced cell proliferation. SELENOS also participates in the early stages of AS, notably by enhancing endothelial function, curbing the expression of adhesion molecules, and lessening leukocyte recruitment—actions that collectively reduce the formation of foam cells. Furthermore, SELENOS forestalls the apoptosis of vascular smooth muscle cells (VSMCs) and macrophages, mitigates vascular calcification, and alleviates inflammation in macrophages and CD4^+^ T cells. These actions help stifle the creation of unstable plaque characterized by thinner fibrous caps, larger necrotic cores, heightened inflammation, and more extensive vascular calcification—features seen in advanced atherosclerotic lesion development. Additionally, serum SELENOS could function as a potential biomarker, and SELENOS single nucleotide polymorphisms (SNPs) rs4965814, rs28628459, and rs9806366, might be effective gene markers for atherosclerosis-related diseases in diabetes. This review accentuates the pathophysiological processes of atherosclerosis in diabetes and amasses current evidence on SELENOS's potential therapeutic benefits or as predictive biomarkers in the various stages of diabetes-induced atherosclerosis.

## Highlights

- SELENOS serves dual roles as potential therapeutics and pivotal biomarkers in various stages of diabetes-induced AS.
- SELENOS plays a significant part in mitigating the initial event of diabetes-induced AS, thereby improving endothelial dysfunction.
- SELENOS actively participates during early AS stages, leading to a decrease in foam cell formation.
- SELENOS aids in the prevention of unstable plaque formation during the progression of advanced atherosclerotic lesions.
- Serum SELENOS and its associated SNPs emerge as promising biomarkers for AS-related diseases in diabetic patients.

## Background

The macrovascular complications associated with diabetes mellitus (DM) present as an accelerated form of atherosclerosis (AS), which subsequently culminates in atherosclerotic cardiovascular disease (ASCVD). ASCVD, characterized as cerebrovascular disease, coronary heart disease (CHD), or peripheral arterial disease presumed to be of atherosclerotic origin, is the dominant cause of morbidity and mortality among diabetic individuals [1, 2]. Recent data from the Heart Disease and Stroke Statistics showed the most common ASCVD complications for those with diabetes to be peripheral artery disease (16.2%) and heart failure (14.1%), followed by stable angina (11.9%), nonfatal myocardial infarction (11.5%), and stroke (10.3%) [3]. Specifically, patients with type 2 DM (T2DM) experience a twofold to fourfold increase in the risk of ASCVD compared to their non-DM counterparts [2, 4]. Additionally, recent research indicates that diabetic patients with intermediate coronary artery stenosis (50–69% stenosis) have poorer clinical outcomes than those with severe coronary artery stenosis (70–100% stenosis) alone [5]. Hence, a thorough comprehension of the pathophysiological pathways and mechanisms instigated by diabetes-induced AS, coupled with the exploration of emerging factors, can facilitate the identification of key biomarkers for early detection and the formulation of novel anti-atherosclerotic drugs.

Selenoprotein S (SELENOS, alternatively known as Tanis, VIMP, AD-015, SEPS1 or SelS) was first identified in the T2DM and metabolic syndrome animal model Psammomys obesus by Walder et al. [6]. The researchers discovered that SELENOS expression in the liver was reduced in both impaired glucose tolerant (IGT) and type 2 diabetic Psammomys obesus relative to normal glucose tolerant (NGT) littermates [6]. In addition, SELENOS expression showed a positive correlation with the plasma triglycerides (TG) and a negative correlation with circulating insulin concentrations [6]. In human subjects, SELENOS expression level in subcutaneous and omental adipose tissue were elevated in the obese subjects and in T2DM patients, and levels of SELENOS were correlated positively with body mass index (BMI), serum levels of high density lipoprotein cholesterol (HDL-C), TG, and insulin resistance assessed by the homeostasis model assessment (HOMA-IR) [7–9]. Furthermore, serum amyloid A (SAA)—an acute-phase response protein elevated in T2DM and a risk factor for cardiovascular disease [10, 11]—was confirmed to interact with SELENOS through surface plasmon resonance (SPR) analysis [6]. This interaction posits a mechanistic link between SELENOS and diabetes-induced AS. Our research group subsequently established that serum SELENOS levels in T2DM patients complicated with subclinical AS and AS patients were significantly elevated compared to those in isolated T2DM subjects. A positive interaction effect was observed between T2DM and AS on serum SELENOS level [12]. From these findings, it is plausible to hypothesize that SELENOS could serve as a novel and optimal target for preventing and managing macrovascular complications in T2DM. The goal of this review is to offer a comprehensive analysis of the existing information on the role of SELENOS in various pathophysiological processes associated with atherosclerosis prompted by diabetes. Our objective is to aid the identification of fundamental biomarkers suitable for clinical application in diagnosing, predicting, and tracking diabetes mellitus patients at a high risk of cardiovascular disease development. Concurrently, we aim to formulate novel therapeutic approaches.

### Pathophysiological processes of atherosclerosis in diabetes mellitus

**Fig. 1:**
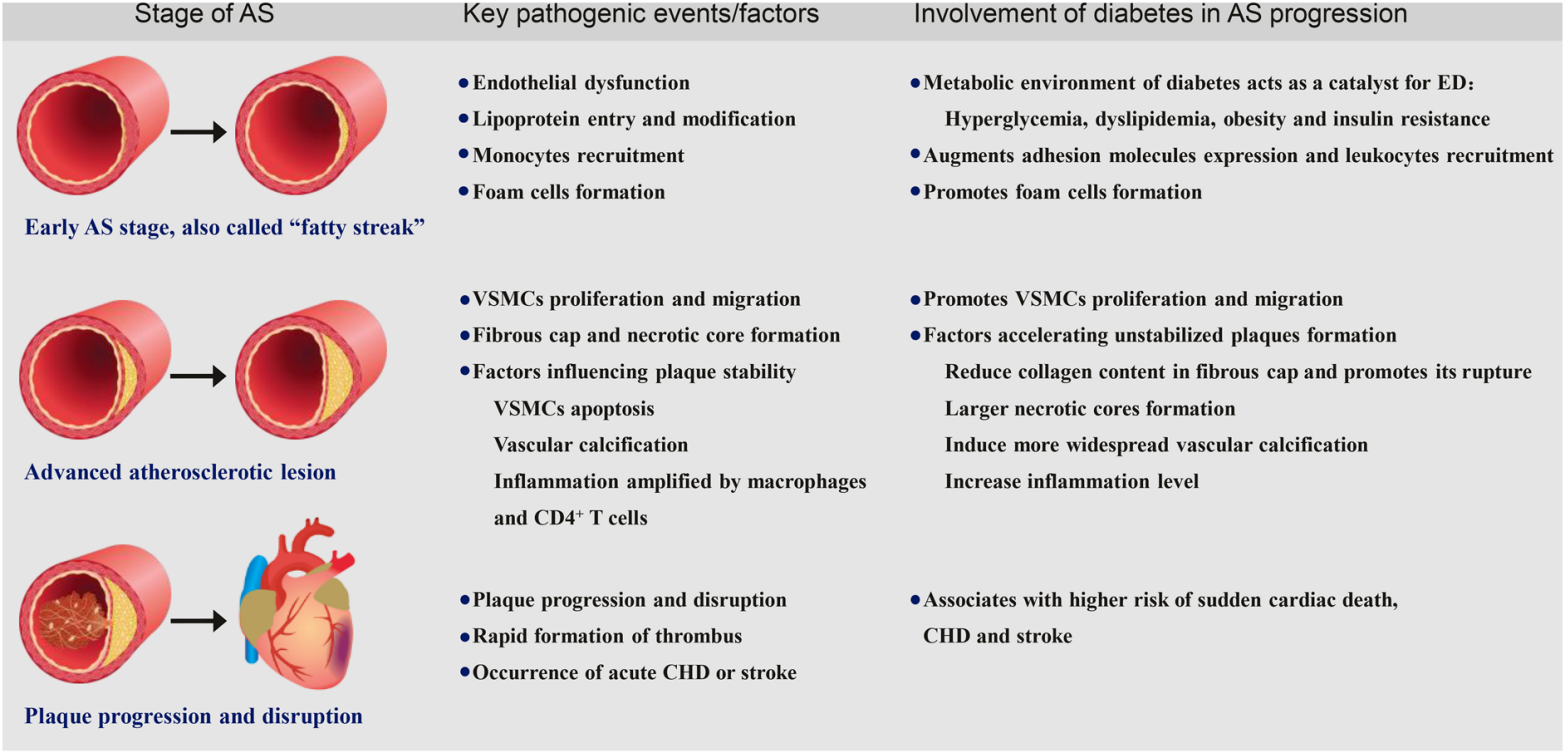
Pathophysiological processes of AS and involvement of diabetes in AS progression. *AS* Atherosclerosis, *ED* Endothelial dysfunction, *VSMCs* Vascular smooth muscle cells, *CHD* Coronary heart disease

AS is a chronic inflammatory disease characterized by progressive arterial wall thickening [13]. DM is associated with the accelerated development of AS. The metabolic environment of T2DM, encompassing hyperglycemia, dyslipidemia, insulin resistance (IR), and obesity, acts as a catalyst for this process [14, 15]. These factors incite ED, a foundational event in atherogenesis (Fig. 1). This disruption results in disturbed vascular homeostasis, manifested by an uptick in vasoconstrictors such as reactive oxidative species (ROS), endothelin-1 (ET-1), and angiotensin II, coupled with a diminution in vasodilators, including prostacyclin (PGI2) and nitric oxide (NO) [16–18].

The transition from normal vascular physiology to ED in the context of diabetes is overseen by several molecular mechanisms. Oxidative stress, often linked to hyperglycemia-associated ED, is stimulated by the escalated production of ROS and reactive nitrogen species (RNS), the accumulation of intracellular advanced glycation end products (AGEs), amplified expression of AGE receptors (RAGE), increased activation of polyol and hexosamine pathways, and the activation of the protein kinase C (PKC) pathway [16, 19]. Hyperglycemia also triggers the activation of nuclear factor-κB (NF-κB), inducing low-grade vascular inflammation [16].

IR, potentially induced by obesity, dyslipidemia, and increased levels of free fatty acids, impairs the phosphatidylinositol-3-kinase (PI3K)-dependent pathway, resulting in decreased NO production. Concurrently, the mitogen-activated protein kinase (MAPK) dependent pathway is activated, leading to an increase in ET-1 production, ultimately triggering ED [16, 20, 21]. Furthermore, recent studies have identified c-jun N-terminal kinase (JNK)-mediated endothelial apoptosis, instigated by high glucose or free fatty acids, as a critical regulator of metabolic dysfunction with potential significance to vascular dysfunction [22, 23]. The endoplasmic reticulum (ER) stress, linked with vascular ED, includes the activation of the inositol requiring enzyme 1 (IRE1) and activating transcription factor 6 (ATF6) pathways, and the induction of activating transcription factor 4 (ATF4) [24–26].

When endothelial cells are activated, there's an upsurge in the expression of adhesion proteins on the cell surface, including vascular cell adhesion molecule-1 (VCAM-1), P-selectin, and intercellular adhesion molecule-1 (ICAM-1). These proteins facilitate the recruitment of monocytes and lymphocytes. Furthermore, there is an escalated discharge of cytokines like matrix metalloprotease-9 (MMP-9) and monocyte chemoattractant protein-1 (MCP-1), which intensifies the migration of monocytes into the sub-endothelial layer of the vessel wall [27–29]. Wan et al. showed augmented intima expression of adhesion molecules ICAM-1 and VCAM-1 in diabetic ApoE^−/−^ mice compared to ApoE^−/−^ mice, and in high glucose-induced human umbilical vein endothelial cells (HUVECs) in vitro, which eventually accelerated atherosclerotic lesions [30] (Fig. 1).

Within the intima, monocytes differentiate into macrophages, a process encouraged by macrophage colony-stimulating factor (M-CSF) and tumor necrosis factor-α (TNF-α). These macrophages participate in the uptake of low-density lipoprotein (LDL) and oxidized-LDL (ox-LDL) particles via phagocytosis, leading to the formation of foam cells, which are present at all stages of lesion development. These macrophage foam cells intensify the inflammatory reaction by discharging cytokines such as interleukin-1 (IL-1) and TNF-α [27]. Moreover, recent reports suggest that at least 50% of the foam cells originate from vascular smooth muscle cells (VSMCs) that have undergone a phenotypic switch to macrophage-like cells within the atherosclerotic plaques [31]. The gathering of these foam cells within the arterial intima results in the creation of what's known as a "fatty streak", which represents the early stage of AS [27–29]. However, persistent oxidative stress induced by hyperglycemia can promote the oxidation of LDL, as well as elevates the formation of AGEs which promotes ox-LDL receptor expression in endothelial cells, thereby promoting ox-LDL production and uptake [32, 33]. Furthermore, reduction in cholesterol efflux induced by AGEs through modulating ATP-binding cassette transporters A1 (ABCA1) expression, transporters involved delivering the excess cholesterol to apo A-I and high-density lipoprotein (HDL), thus leading to intracellular lipid accumulation in macrophage foam cells [34]. (Fig. 1).

If the endothelium continues to be chronically injured, fatty streaks may evolve into advanced atherosclerotic lesions. In this transition, VSMCs proliferate and migrate from the tunica media to the intima, synthesizing and secreting extracellular matrix proteins and collagen. This activity contributes to the formation of a fibrous cap over the plaque. Lipid-engorged foam cells, whether derived from macrophages or VSMCs, eventually undergo apoptosis, thereby contributing to the expansion of an acellular area within the lesion, known as the necrotic core. The plaques can develop a stable fibrous cap, isolating them from the vessel environment. Plaque destabilization can occur due to the erosion or rupture of the fibrous cap, driven by matrix metalloproteinases (MMPs). These enzymes, secreted by macrophage/foam cells, promote extracellular matrix degradation, which can ultimately trigger platelet aggregation and thrombosis [27, 28]. However, hyperglycemia promoted the proliferation and migration of VSMCs in diabetic mice and high glucose-stimulated human aortic vascular smooth muscle cells (HA/VSMCs), meanwhile, the expression and activity levels of MMPs were significantly increased [35]. Furthermore, Wan et al. demonstrated that the collagen content percentage in atherosclerotic plaque drastically decreased in diabetic ApoE-/- mice, indicating more unstabilized plaques formation in diabetes-induced AS [30] (Fig. 1).

The stability of atherosclerotic plaques is significantly influenced by the thickness of the fibrous cap, the volume of the necrotic core, and the degree of inflammation inside the fibrous cap. Factors such as VSMC apoptosis, vascular calcification, and the inflammatory response amplified by macrophage foam cells play crucial roles. Moreover, there is an accumulation of CD4^+^ T cells within the expanding lesion, which adds to the local inflammatory atmosphere by producing pro-inflammatory cytokines [27]. As the fibrous cap thins and the necrotic core expands, the plaque becomes unstable or vulnerable [36]. Significantly, plaques in the coronary arteries of diabetic patients exhibit larger necrotic cores and considerably increased inflammation compared to those in non-diabetic individuals. Furthermore, patients with type 2 diabetes mellitus display more widespread vascular calcification in coronary, carotid, and other arterial regions [37] (Fig. 1).

Atherosclerotic plaques can gradually narrow the lumen of the blood vessel, impairing blood flow and leading to chronic ischemia. However, a far greater danger lies in the rapid thrombus formation, triggered by plaque erosion or rupture, which can abruptly block blood flow and cause fatal myocardial infarction or stroke [27, 28, 38]. Evidence indicated that diabetes was associated with a higher risk of sudden cardiac death (HR = 2.18, 95% CI 1.89–2.52), incident CHD events (RR = 2.82, 95% CI 2.35–3.38) and stroke (RR = 2.28, 95% CI 1.93–2.69) [39]. (Fig. 1).

### SELENOS effects involved in diabetes-induced atherosclerosis

#### Pathophysiological role of SELENOS in biological systems

SELENOS was initially confirmed to be a receptor for the acute inflammatory response protein, SAA [6], and there has been an NF-κB binding site within the -601–398 region of SELENOS gene promoter, recognized as a positive regulatory element for regulation of SELENOS expression [40]. In addition, SELENOS was proved to be a thioredoxin-dependent reductase, which was exerted through 188th selenocysteine and maintained through the restoration of selenosulfide bond between 188th selenocysteine and 174th cysteine [41, 42]. Moreover, researchers also found that SELENOS had peroxidase activity, that could break down hydrogen peroxide (H_2_O_2_) into H_2_O [42]. Furthermore, as an ER membrane protein, SELENOS exerts a significant role in sustaining the morphology and distribution of ER [43], and forms a complex composed of degradation in ER protein 1 (Derlin1)-ubiquitin ligase E3-p97ATPase-SELENOK for degrading unfolded or misfolded proteins in the ER, which is known as ER-associated protein degradation (ERAD) process [44–46].

Thus, SELENOS is involved in the pathophysiological regulation of inflammation, oxidative stress, and ER stress, indicating a tightly relationship of SELENOS with the occurrence and development of DM and macrovascular complications.

#### SELENOS ssociated with pathogenic factors for endothelial dysfunction

Research on the polygenic animal model of type 2 diabetes, Psammomys obesus, has shown that the expression of SELENOS in the liver is inversely correlated with circulating glucose and insulin levels and directly proportional with plasma TG concentrations [6]. In hepatoma H4IIE cells, overexpression of SELENOS led to decreased hepatic glucose utilization by reducing glucose uptake, glycogen synthesis and content. It also mitigated the suppressive effect of insulin on gluconeogenesis, leading to increased hepatic glucose output [47].

Interestingly, SELENOS was secreted from hepatoma HepG2 cells, but not from a variety of other examined cell types. These include human embryonic kidney 293 cells, kidney Cos7 cells, 3T3-L1 pre-adipocytes, skeletal muscle L6 cells, macrophage RAW264.7 cells, HUVECs, and HA/VSMCs [12, 48]. When serum SELENOS levels were measured in healthy human subjects and those with type 1 and type 2 diabetes, the protein was detected in 65 out of 209 subjects, a detection rate of 31.1%. The average levels of these positive subjects across the three groups were not statistically different [48].

However, these results appear inconsistent with other research. In a previous study, all tested subjects (100%) showed detectable serum SELENOS, and levels in type 2 diabetic patients were lower compared to healthy controls. In this research, there was a negative correlation observed between SELENOS levels and waist circumference (WC), as well as fasting plasma glucose (FPG) [12]. Consistently, another study found lower serum SELENOS in metabolic syndrome patients and cardiovascular disease patients compared to patients without metabolic syndrome, and a negative relationship was noted between the levels of SELENOS, WC, and fasting blood sugar (FBS) [49]. The disparities could possibly be attributed to the variations in race and geographic location of the human participants involved in the studies, as well as the different enzyme-linked immunosorbent assay (ELISA) systems employed [12, 48, 49]. The connection between SELENOS, LDL, and very low-density lipoprotein (VLDL) was suggested in Gao's study, which found SELENOS in human serum fractionated into HDL, LDL, and VLDL [48]. There was also a positive correlation found between SELENOS levels and HDL [49]. In line with these findings, a study on apolipoprotein E deficient (ApoE^−/−^) mice suggested that hepatic SELENOS might be associated with dyslipidemia, as selenium nanoparticles (SeNPs) significantly decreased total cholesterol (TC), TG, and Low-density lipoprotein cholesterol, L (LDL-C) levels and increased serum HDL-C. Additionally, SeNPs enhanced the expression levels of SELENOS in the liver [50].

In Swedish obese subjects, with an average BMI of 37.7 kg/m^2^, SELENOS gene expression in subcutaneous adipose tissue was higher than in lean counterparts, who exhibited an average BMI of 22.0 kg/m^2^. Furthermore, in these obese subjects, SELENOS expression level correlated positively with BMI, fat mass, serum levels of HDL-C, TG, and HOMA-IR. Additionally, a positive correlation was noted between SELENOS expression and waist circumference, as well as fat-free mass [7]. Echoing these findings, our team recently established that in human subjects, levels of SELENOS in both subcutaneous and omental fat were elevated in the obese group (BMI ≥ 28.0 kg/m^2^) compared to the non-obese group (BMI < 28.0 kg/m^2^) [8]. Furthermore, our research identified SELENOS as a positive regulator for the process of fat cell differentiation, known as adipogenesis, in 3T3-L1 preadipocytes. This regulation occurs through the IRE1α-X-box-binding protein 1 (XBP1) pathway [8, 51].

However, other studies have pointed to an anti-adipogenic role of SELENOS. Reduction in SELENOS expression, mediated by peroxisome proliferator-activated receptor γ (PPARγ)-induced ubiquitination, was shown to promote adipocyte differentiation, potentially through modulation of ER stress and its related ubiquitin–proteasome system (UPS) [52–54].

The absence of impact on obesity and body composition observed in SELENOS knockout mice sharply contrasts with the findings in both in-vivo adipose tissue and in-vitro 3T3-L1 preadipocytes. In SELENOS-deficient (SELENOS^−/−^), heterozygous (SELENOS^−/+^), and wild-type mice, neither genetic reduction nor deletion of SELENOS had any notable impact on whole-body metabolism, body weight, fat mass, or lean mass. These mice displayed similar oxygen consumption (VO_2_), carbon dioxide production (VCO_2_), and respiratory exchange ratio (RER), as measured using metabolic cages. Furthermore, body composition, which is determined by magnetic resonance imaging (MRI), was also alike across all groups [55, 56].

The discrepancies between these findings underscore the complexity of deciphering SELENOS function and highlight the necessity for further in-vivo and in-vitro studies. Considering that SELENOS performs diverse biological functions across different tissues and organs [57], it might explain why no discernible differences were observed in global SELENOS knockout mice. Thus, the creation of tissue-specific knockout mice could provide more in-depth insights into SELENOS's role. To this end, our group recently engineered hepatic-specific SELENOS knockout mice (SelS^H−KO^). Indeed, our findings indicated that hepatic-specific deletion of SELENOS accelerated the onset and progression of obesity, impaired glucose tolerance and insulin sensitivity, and increased hepatic TG and diacylglycerol (DAG) accumulation. This seemed to be regulated by encouraging fatty acid absorption and lessening fatty acid oxidation [58].

The compromised function of pancreatic β-cells and a decrease in β-cell mass, frequently as a result of excessive β-cell apoptosis and reduced β-cell proliferation, are fundamental factors contributing to insulin resistance and the emergence of T2DM [59, 60].

Overexpression of SELENOS was shown to protect Min6 β-cells, a mouse insulinoma cell line, from oxidative stress-induced apoptosis, suggesting that SELENOS could be important for insulin secretion and insulin sensitivity [61]. On the other hand, the knockdown of SELENOS in Min6 cells induced β-cell apoptosis and reduced cell proliferation. This effect was associated with a decrease in the activation of the unfolded protein response (UPR), ultimately leading to the endoplasmic reticulum (ER) stress [62].

However, it appears confusing that SELENOS knockdown increased insulin production and secretion in Men's study. The researchers speculated that this might be a feedback reaction to the decline in cell survival and proliferation, considering that insulin is essential for β-cell survival and proliferation [62]. Furthermore, malfunction of pancreatic β-cells can be triggered by a flaw in insulin signaling within the β-cells, which leads to β-cell insulin resistance [63]. Hence, it's plausible to theorize that the knockdown of SELENOS might instigate β-cell insulin resistance, necessitating a compensatory rise in insulin secretion. This, in turn, could result in the observed escalation in insulin production and secretion.

Accumulation of lipids, particularly saturated fatty acids, in the liver, adipose tissue, and skeletal muscle, has been associated with IR and T2DM. Several mechanisms have been implicated in this process, including oxidative stress, inflammatory signaling, ER stress, and cell death [64–66]. In a hepatic steatosis model using pigs, selenium supplementation alleviated oxidative damage and apoptosis induced by a high-fat diet (HFD), alongside an increase in SELENOS expression in the liver [67]. Furthermore, the silencing of SELENOS via small interference RNA (siRNA) was found to significantly exacerbate the inflammatory response, apoptosis, and oxidative stress in hepatoma HepG2 and Hepa1-6 cells induced by β-mercaptoethanol (an ER stress agent) and lipopolysaccharide (LPS) [68–70]. Conversely, the overexpression of SELENOS in HepG2 cells was observed to mitigate ER stress and reduce NF-κB activity [71].

Our group demonstrated that SELENOS mRNA expression in human omental adipose tissues was higher in individuals with T2DM than in those without the condition, with SELENOS levels positively correlated with HOMA-IR [9]. Furthermore, SELENOS knockdown in murine C2C12 myoblasts decreased cell viability and exacerbated ER and oxidative stress responses in the presence of palmitate, suggesting a role for SELENOS in skeletal muscle insulin resistance [72].

Genetic polymorphisms of SELENOS, such as single nucleotide polymorphisms (SNPs), have been linked with metabolic disorders and DM. For instance, the SELENOS SNP rs4965373 was linked to increased serum insulin levels and HOMA-IR in a cohort of 618 Swedish patients with acute coronary symptoms and 618 healthy controls [7]. Another SNP, rs12910524, was discovered to be linked with increased TG concentrations in both Han and Uygur ethnic groups of nondiabetic Chinese subjects, even after adjusting for sex, age, alcohol intake, smoking, BMI, and plasma glucose levels [73].

In a study by Zhao et al. [74] comprising 1947 T2DM patients and 1639 control subjects, four SELENOS SNPs were genotyped (rs12910524, rs1384565, rs2101171, rs4965814), and rs1384565 was found to be an independent risk factor for T2DM in a Chinese population.

However, other studies have yielded negative results. No significant differences were observed in the SELENOS SNPs (rs28665122 and rs4965373) between subjects with metabolic syndrome (n = 71) and without metabolic syndrome (n = 65) in an Iranian population [75]. In a similar vein, there was no significant disparity in the genotype and allele distribution of SELENOS SNPs (rs4965814, rs28665122, rs34713741, and rs4965373) between type 2 diabetes mellitus patients (n = 170) and healthy controls (n = 100) in a Chinese population [76]. Additionally, no association was detected between SELENOS SNPs (rs11327127, rs28665122, rs4965814, rs12917258, rs4965373, and rs2101171) and type 1 DM (n = 311) compared to healthy controls (n = 550) in Spanish subjects [77, 78].

It is important to consider that factors such as ethnicity, sex, age, SNP genotyping methods, and the number of subjects can confound results from genome-wide association studies, potentially leading to discrepancies between studies. Therefore, to further elucidate the relationship between SELENOS gene variation and the risk of metabolic diseases, more studies should be conducted, ideally using stratified sub-group analysis and larger cohorts. Further investigation is also needed to understand the mechanisms that link SELENOS SNPs to metabolic diseases.

#### SELENOS involved in vascular endothelial dysfunction

SELENOS has been identified as a potential receptor for SAA [6], which is a key player in promoting vascular ED and AS development [11, 79–81]. Studies have shown positive correlations between SELENOS expression in skeletal muscle and adipose tissue and SAA [9, 82].

Atherosclerotic lesions in ApoE^−/−^ mice were found to be alleviated by SeNPs, with increased SELENOS expression observed in the liver [50]. Moreover, the expression of SELENOS was heightened in the vascular wall intima of streptozotocin (STZ)-induced diabetic rats and low-density lipoprotein receptor (LDLR) knockout mice induced by a high-fat diet (HFD) [83, 84]. These findings suggest that SELENOS is involved in vascular ED.

Indeed, an increasing amount of research underscores the protective role of SELENOS in vascular endothelial cells. Our group discovered that overexpressing SELENOS in HUVECs significantly bolstered cell viability and superoxide dismutase (SOD) activity, while simultaneously reducing malondialdehyde (MDA) production and caveolin-1 (Cav-1) expression in response to hydrogen peroxide (H_2_O_2_) treatment. In contrast, the silencing of SELENOS was associated with decreased cell viability, reduced SOD activity, and diminished protein kinase Cα (PKCα) expression, while MDA production and Cav-1 expression were increased [85].

Following this, our study employed an integrated microfluidic chip that was designed to simulate the diabetic vascular endothelial microenvironment. This was established with accurate concentrations of glucose and oxidized-LDL (ox-LDL). We delved deeper into understanding the role and mechanism of SELENOS in oxidative damage to human aortic endothelial cells (HAECs), which was caused by the combined impact of high glucose levels and/or ox-LDL [86].

The results demonstrated that SELENOS provided protection to HAECs against oxidative stress injury induced by multiple factors, evidenced by increased cell viability, reduced ET-1 and reactive oxygen species (ROS) levels, and augmented SOD1 and SOD2 expression. These findings align with our previous study. Further, it was confirmed that the antioxidant protective effect of SELENOS within the diabetic vascular endothelial microenvironment was facilitated through inhibiting PKCα and subsequently activating the PI3K/protein kinase B (Akt)/eNOS signaling pathway [86]. Moreover, SELENOS was shown to protect against endothelial injury in HAECs prompted by high glucose and/or ox-LDL, with the underlying mechanisms potentially associated with its regulation of autophagy through the activation of the Akt/mammalian target of rapamycin (mTOR) signaling pathway [87]. Furthermore, SELENOS inhibited the growth in endothelial apoptosis and cleaved caspase3 levels induced by high glucose, which coincided with the suppression of the PKCβII/JNK/B-cell lymphoma/leukemia-2 (Bcl-2) pathway. The protective effects of SELENOS were countered, and apoptosis and cleaved caspase3 levels increased when HUVECs were pretreated with PKC activators [83]. Additionally, overexpression of SELENOS prevented the reduction of NO and eNOS, as well as the rise of ET-1 and ROS triggered by TNF-α [84].

The levels of TNF-α-induced ICAM-1 and VCAM-1 expression were found to be reduced, along with the adhesion of THP-1 cells to HUVECs. Additionally, there was observed suppression of inflammatory factors, including interleukin-1β (IL-1β), interleukin-6 (IL-6), interleukin-8 (IL-8), and MCP-1. These discoveries imply the potential function of SELENOS in mitigating leukocyte adhesion by suppressing adhesion molecules [84]. Further, overexpression of SELENOS was shown to mitigate TNF-α-induced activation of the MAPK and NF-κB pathways. In contrast, the silencing of SELENOS resulted in amplified TNF-α-induced damage in HUVECs. Aligning with our results, the suppression of SELENOS significantly induced an inflammatory response as the expression levels of TNF-α and IL-1β were elevated in arterial endothelial cells, and enhanced neutrophil adhesion was observed [88].

In conclusion, SELENOS appears to be a promising contender for the early prevention and management of macrovascular complications associated with diabetes.

#### SELENOS effect on the advanced atherosclerotic lesion

VSMCs have a notable role in the formation and structure of advanced AS lesions, as well as in vascular calcification [36, 89]. Both intimal and medial calcification in arteries, primarily driven by VSMCs, are associated with atherosclerotic plaque rupture and vessel stiffness [36, 89]. Additionally, apoptosis of VSMCs contributes to the destabilization and rupture of atherosclerotic plaques and promotes vascular calcification [36, 89].

A study by Ye et al. demonstrated that silencing SELENOS through siRNA makes VSMCs more susceptible to oxidative injury and apoptosis, triggered by H_2_O_2_ or tunicamycin [90]. It also enhances the phosphorylation of MAPK and JNK in VSMCs. Moreover, SELENOS silence exacerbates ER stress induced by H2O2 or tunicamycin, as indicated by elevated protein levels of ER stress transducer phosphorylated protein kinase RNA-like ER kinase (PERK), ER chaperone glucose-regulated protein 78 (GRP78), and the proapoptotic transcription factor CCAAT/enhancer-binding-protein (C/EBP) homologous protein (CHOP) [90].

Furthermore, they investigated SELENOS's role in inflammation-induced vascular calcification. SELENOS knockdown worsened LPS- or TNF-α-induced osteoblastic differentiation and calcification of VSMCs. This was evidenced by the increased levels of key osteogenic transcription factors like bone-related proteins and runt-related transcription factor 2 (Runx2), including alkaline phosphatase and type I collagen, along with calcium deposition content. Both the classical and alternative pathways of NF-κB signaling were activated, with increases in ER stress markers GRP78 and IRE1α expression observed in calcifying VSMCs [91].

These findings provide new insights into SELENOS's effect on VSMCs apoptosis and vascular calcification, which could be potentially beneficial for preventing and treating ASCVD.

Macrophages and CD4^+^ T cells play pivotal roles in the inflammatory response seen throughout all stages of atherosclerotic lesion development [27–29]. The stimulation of macrophages with LPS is frequently used as an effective model for studying inflammatory responses and for evaluating potential anti-inflammatory agents [92, 93].

SELENOS has been linked to inflammation induced by LPS-stimulated RAW264.7 macrophages. Specifically, it has been observed that selenium pretreatment alleviated immunological stress in these cells, reducing inflammation cytokines such as IL-6, IL-1β, IL-10, TNF-α, and MCP-1, while simultaneously increasing SELENOS expression [94].

In advanced atherosclerotic lesions, the apoptosis of lipid-engorged foam cells, whether originating from macrophages or VSMCs, contributes to the generation and development of the pro-inflammatory necrotic lipid core [95]. Kim et al. discovered that overexpression of SELENOS protected RAW264.7 macrophages against ER stress-induced cytotoxicity and apoptosis, thereby promoting cell survival [96]. In contrast, suppression of SELENOS sensitized cells to ER stress-induced cell death. These findings suggest that SELENOS could be a promising therapeutic target for atherosclerosis.

Furthermore, SELENOS has been identified as a gene regulating the effector functions of CD4^+^ T cells. After SELENOS knockdown, increased levels of IL-2, IL-21, and granulocyte–macrophage colony-stimulating factor (GM-CSF) were observed in the culture media. This effect was found to be regulated via both the early 2 factor (E2F) transcription factor 5 (E2F5) regulatory pathway and the Ca^2+^/ immune transcription factor nuclear factor of activated T cells, cytoplasmic 2 (NFATC2) signaling pathway [97]. This adds another layer of complexity to our understanding of the role of SELENOS in immune responses and inflammation.

#### SELENOS may act as a biomarker for atherosclerosis

SELENOS has been identified in the serum of human subjects, and its relationships with subclinical atherosclerosis (SAS) and AS have been explored [12, 48, 49]. Our group found that there was no notable disparity in serum SELENOS levels among non-diabetic groups (including healthy controls, isolated SAS, and isolated AS groups), while the levels of SELENOS increased when T2DM was complicated by either SAS or AS. Specifically, serum SELENOS levels were higher in groups with T2DM complicated by SAS (DSAS) and T2DM complicated by AS (DAS) than in the isolated T2DM group. However, there was no significant difference between the DSAS and DAS groups [12].

These discoveries imply that serum SELENOS could potentially act as a biomarker, potentially a risk factor, for the development of AS caused by diabetes. Moreover, SELENOS may serve as a novel and optimal target for managing macrovascular complications in T2DM.

#### SELENOS SNPs maybe gene markers for atherosclerosis

Multiple studies have suggested a significant correlation between SELENOS SNPs and susceptibility to atherosclerosis-related diseases. These findings suggest that SELENOS gene polymorphisms could serve as genetic markers for predicting the risk of atherosclerosis.

Moreover, an increasing amount of research has unveiled a significant correlation between SELENOS SNPs and susceptibility to AS-related diseases. This suggests that SELENOS gene polymorphisms might serve as promising genetic markers for predicting the risk of AS. For instance, in a case–control study composed of 2,222 subjects from the FINRISK Study in Finland, the SELENOS SNP rs8025174 was projected to enhance the risk of CHD in females by 2.95 times [98]. Furthermore, the SNP rs7178239 was found to elevate the risk of ischemic stroke by 1.75 times across both genders and 3.35 times in females [98]. However, no connection was observed between the SNP rs28665122 and CHD or ischemic stroke [98, 99]. Subsequent studies discovered that carrying the SELENOS SNP rs4965814 and rs9874 escalated the risk of ischemic stroke in Finnish women by 2.89 and 3.32 times, respectively [100]. These findings were echoed by Li et al. and Qiu et al. [101, 102], who found that the SELENOS SNP rs4965814 could amplify the risk of ischemic stroke by 1.54 times in both genders and 2.43 times in females within a Chinese sample population of 239 ischemic stroke patients and 240 non-ischemic stroke control subjects. (Table 1).

**Table 1:** Association between SELENOS SNPs and the risk of metabolic disorders, diabetes and AS-related diseases

No	Rs number^a^	Position^a^	Polymorphism [1/2]	Risk of related disorders and diseases	References
1	rs28665122	Chr15:101277522	C/T	Subclinical CVD in T2DM	[104]
2	rs8025174	Chr15:101279548	C/A	Coronary heart disease	[98]
3	rs4965814	Chr15:101273712	T/C	Ischemic stroke	[100–102]
			T/C	Subclinical CVD in T2DM	[104]
			T/C	CVD in T2DM	[104]
4	rs12917258	Chr15:101273134	G/C	Subclinical CVD in T2DM	[104]
5	rs4965373	Chr15:101272190	G/A	Serum insulin, HOMA-IR	[7]
6	rs9874	Chr15:101271199	T/C	Ischemic stroke	[100]
7	rs28628459	Chr15:101272152	T/C	Subclinical CVD in T2DM	[104]
				CVD in T2DM	[104]
8	rs7178239	Chr15:101267907	C/G	Ischemic stroke	[98]
				Subclinical CVD in T2DM	[104]
9	rs9806366	Chr15:101262752	C/T	CVD in T2DM	[104]
10	rs12910524	Chr15:101262360	C/T	TG concentration	[73]
11	rs1384565	Chr15:101264707	T/C	T2DM	[74]

*Chr* Chromosome, *CVD* Cardiovascular disease, *T2DM* Type 2 diabetes mellitus

1 = major allele, 2 = minor allele

^a^Rs numbers and position information are from the PubMed SNP database

However, in a case–control study conducted in Germany, which comprised 470 ischemic stroke patients and 807 population controls, no significant interaction effects of the SELENOS SNP rs9874 were found [103]. This discrepancy may be partially due to the absence of sex-stratified sub-group analysis in the study. Further, Cox et al. examined the correlation between ten types of SELENOS SNPs and the risk of AS in T2DM patients, using a sample of 1220 European American T2DM subjects from the Diabetes Heart Disease Study [104]. This study discovered that the SELENOS SNPs rs28665122, rs4965814, rs28628459, rs7178239, and rs12917258 were associated with SAS, while the SNPs rs4965814, rs28628459, and rs9806366 were associated with clinical AS. Additionally, the SELENOS SNP rs34713741 was linked to a 1.49-fold increase in the risk of PAD among Polish subjects (PAD group n = 664, control group n = 543) [105]. Recently, Wang et al. reported that the SELENOS SNP rs117613208 raised the risk of coronary artery disease (CAD) by 2.107-fold in a Chinese population-based case–control study (576 CAD cases and 452 control subjects) [106]. Furthermore, leveraging this locus, they developed a diagnostic model for CAD, referred to as the GASDLY score. This model exhibited a sensitivity of 74.7% and a specificity of 75.5%. The GASDLY score is calculated using the following formula: GASDLY score = −2.145 + (age × 0.59) + (smoking × 1.675) + (diabetes × 0.724) + (rs117613208 TT genotype × 0.745) + (lipoprotein A × 0.002)—(1.817 × apolipoprotein A1) [106]. (Table 1).

These findings suggest that SELENOS gene polymorphisms may serve as valuable genetic indicators for screening and evaluating the risk of macroangiopathy in both non-diabetic and T2DM patients.

## Conclusions and future perspectives

In conclusion, SELENOS performs a complex and multifaceted function in the pathophysiology of atherosclerosis induced by diabetes.

**Fig. 2:**
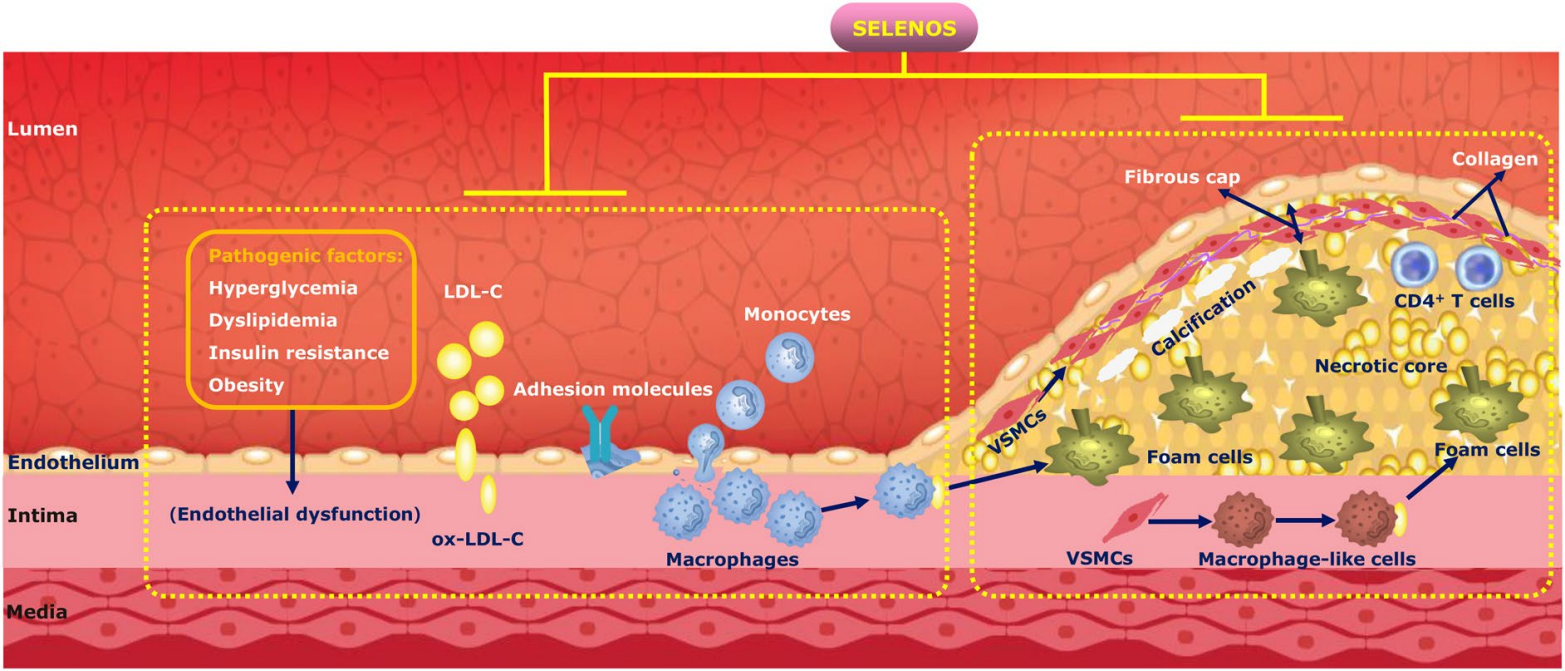
SELENOS role in the pathophysiological processes of diabetes-induced AS. In the early AS stage, SELENOS was associated with pathogenic factors for ED, including hyperglycemia, dyslipidemia, insulin resistance and obesity, though the double-sided effects and inconsistent results were shown, which requires further studies to draw the conclusion. Moreover, SELENOS could ameliorate endothelial function, reduce adhesion molecules expression and leukocytes recruitment giving rise to the reduction of foam cells formation. During the advanced atherosclerotic lesion development, SELENOS prevented VSMCs and macrophages apoptosis, reduced vascular calcification, and alleviated inflammation in macrophages and CD4 + T cells, which contributed to inhibiting the formation of unstable plaque characterized by a thinner fibrous cap, larger necrotic core, greater inflammation and more extensive vascular calcification. *SELENOS* Selenoprotein S, *LDL-C* Low-density lipoprotein cholesterol, *ox-LDL-C* Oxidized LDL-C, *VSMCs* Vascular smooth muscle cells

At the early stages of atherosclerosis, SELENOS is linked to key pathological factors, including hyperglycemia, dyslipidemia, obesity, and insulin resistance (Fig. 2). However, the effects of SELENOS at this stage are double-sided, and the results across different studies are inconsistent, necessitating further research to establish a definitive understanding of SELENOS effect on diabetes and its related conditions, which may provide a new intervention target or anti-diabetic strategy for the prevention and treatment of DM.

Additionally, SELENOS seems to exert a protective effect on endothelial function, reduces the expression of adhesion molecules and leukocyte recruitment, and minimizes the formation of foam cells (Fig. 2). In the context of advanced atherosclerotic lesions, SELENOS appears to mitigate apoptosis in VSMCs and macrophages, reduce vascular calcification, and decrease inflammation in macrophages and CD4^+^ T cells (Fig. 2). This could potentially inhibit the formation of unstable plaques characterized by thin fibrous caps, larger necrotic cores, more extensive inflammation, and extensive vascular calcification. Thus, it is necessary to design and synthesize SELENOS in vitro, and further investigate its role and mechanism on diabetes-induced atherosclerosis, which may serve as a novel therapeutic for the treatment of diabetic macroangiopathy in the future.

Nevertheless, the exact roles and mechanisms of SELENOS in the processes of plaque erosion and rupture remain elusive due to the difficulty in modeling these processes in atherosclerotic animals in vivo or cells in vitro. As such, the roles of SELENOS in these processes are mainly discussed in the context of retrospective case–control studies.

Given the reported links between serum SELENOS levels and SNPs in the SELENOS gene with diabetes-induced atherosclerosis, SELENOS may act as a potential biomarker and its gene variations as promising genetic indicators for the evaluation of the risk of macroangiopathy in non-DM and DM patients, and suggesting that early enhanced primary prevention measures should be applied to the population carrying relevant SNPs. However, these possibilities require further case-controlled studies that involve more SELENOS SNPs, different races, and larger sample sizes.

## Data Availability

Not applicable.

## Abbreviations

DM: Diabetes mellitus
AS: Atherosclerosis
ASCVD: Atherosclerotic cardiovascular disease
CHD: Coronary heart disease
T2DM: Type 2 DM
SELENOS: Selenoprotein S
IGT: Impaired glucose tolerant
NGT: Normal glucose tolerant
SAA: Serum amyloid A
SPR: Surface plasmon resonance
IR: Insulin resistance
ED: Endothelial dysfunction
ET-1: Endothelin-1
ROS: Reactive oxidative species
NO: Nitric oxide
PGI2: Prostacyclin
RNS: Reactive nitrogen species
AGEs: Advanced glycation end products
RAGE: AGEs receptors
PKC: Protein kinase C
NF-κb: Nuclear factor-κB
PI3K: Phosphatidylinositol-3-kinase
MAPK: Mitogen activated protein kinase
JNK: C-jun N-terminal kinase
IRE1: Inositol requiring enzyme 1
ATF6: Activating transcription factor 6
ATF4: Activating transcription factor 4
VCAM-1: Vascular cell adhesion molecule-1
ICAM-1: Intercellular adhesion molecule-1
MCP-1: Monocyte chemoattractant protein-1
MMP-9: Matrix metalloprotease-9
M-CSF: Macrophage colony-stimulating factor
TNF-α: Tumor necrosis factor-α
IL-1: Interleukin-1
VSMCs: Vascular smooth muscle cells
MMPs: Matrix metalloproteinases
TG: Triglyceride
DAG: Diacylglycerol
HUVECs: Human umbilical vein endothelial cells
HA/VSMCs: Human aortic vascular smooth muscle cells
WC: Waist circumference
FPG: Fasting plasma glucose
FBS: Fasting blood sugar
ELISA: Enzyme linked immunosorbent assay
HDL: High-density lipoprotein
LDL: Low-density lipoprotein
VLDL: Very low-density lipoprotein
SeNPs: Selenium nanoparticles
TC: Total cholesterol
LDL-C: Low-density lipoprotein cholesterol
HDL-C: High-density lipoprotein cholesterol
BMI: Body mass index
HOMA-IR: Homeostasis model assessment of IR
XBP1: X-box-binding protein 1
PPARγ: Peroxisome proliferator-activated receptor γ
UPS: Ubiquitin–proteasome system
VO_2_: Oxygen consumption
VCO_2_: Carbon dioxide production
RER: Respiratory exchange ratio
MRI: Magnetic resonance imaging
KD: Knock down
UPR: Unfolded protein response
ER: Endoplasmic reticulum
HFD: High-fat diet
LPS: Lipopolysaccharide
SNP: Single nucleotide polymorphism
LDLR: LDL receptor
SOD: Superoxide dismutase
MDA: Malondialdehyde
Cav-1: Caveolin-1
H_2_O_2_: Hydrogen peroxide
PKCα: Protein kinase Cα
ox-LDL: Oxidized-LDL
HAECs: Human aortic endothelial cells
Akt: Protein kinase B
mTOR: Mammalian target of rapamycin
Bcl-2: B-cell lymphoma/leukemia-2
IL-1β: Interleukin-1β
IL-6: Interleukin-6
IL-8: Interleukin-8
siRNA: Small interference RNA
GRP78: Glucose regulated protein 78
PERK: Protein kinase RNA like ER kinase
C/EBP: CCAAT/enhancer-binding-protein
CHOP: C/EBP homologous protein
LPS: Lipopolysaccharide
Runx2: Runt-related transcription factor 2
GM-CSF: Granulocyte–macrophage colony-stimulating factor
E2F: Early 2 factor
E2F5: E2F transcription factor 5
NFATC2: Ca^2+^/immune transcription factor nuclear factor of activated T cells, cytoplasmic 2
SAS: Subclinical AS
DSAS: DM complicated with SAS
DAS: DM complicated with AS
PAD: Peripheral arterial disease
CAD: Coronary artery disease
